# The Qatar Biobank: background and methods

**DOI:** 10.1186/s12889-015-2522-7

**Published:** 2015-12-03

**Authors:** Hanan Al Kuwari, Asma Al Thani, Ajayeb Al Marri, Abdulla Al Kaabi, Hadi Abderrahim, Nahla Afifi, Fatima Qafoud, Queenie Chan, Ioanna Tzoulaki, Paul Downey, Heather Ward, Neil Murphy, Elio Riboli, Paul Elliott

**Affiliations:** Hamad Medical Corporation, P O Box 3050, Doha, Qatar; Department of Health Sciences, College of Arts and Sciences, Qatar University, P O Box 348, Doha, Qatar; Sidra Medical and Research Centre, P O Box 26999, Doha, Qatar; Qatar Biobank, Qatar Foundation for Education, Science, and Community, P O Box 5825, Doha, Qatar; School of Public Health, Imperial College London, St Mary’s campus, Norfolk Place, London, UK

**Keywords:** Biobank, Cohort study, Obesity, Population health, Risk factors

## Abstract

**Background:**

The Qatar Biobank aims to collect extensive lifestyle, clinical, and biological information from up to 60,000 men and women Qatari nationals and long-term residents (individuals living in the country for ≥15 years) aged ≥18 years (approximately one-fifth of all Qatari citizens), to follow up these same individuals over the long term to record any subsequent disease, and hence to study the causes and progression of disease, and disease burden, in the Qatari population.

**Methods:**

Between the 11^th^-December-2012 and 20^th^-February-2014, 1209 participants were recruited into the pilot study of the Qatar Biobank. At recruitment, extensive phenotype information was collected from each participant, including information/measurements of socio-demographic factors, prevalent health conditions, diet, lifestyle, anthropometry, body composition, bone health, cognitive function, grip strength, retinal imaging, total body dual energy X-ray absorptiometry, and measurements of cardiovascular and respiratory function. Blood, urine, and saliva were collected and stored for future research use. A panel of 66 clinical biomarkers was routinely measured on fresh blood samples in all participants. Rates of recruitment are to be progressively increased in the coming period and the recruitment base widened to achieve a cohort of consented individuals broadly representative of the eligible Qatari population. In addition, it is planned to add additional measures in sub-samples of the cohort, including Magnetic Resonance Imaging (MRI) of the brain, heart and abdomen.

**Results:**

The mean time for collection of the extensive phenotypic information and biological samples from each participant at the baseline recruitment visit was 179 min. The 1209 pilot study participants (506 men and 703 women) were aged between 28–80 years (median 39 years); 899 (74.4 %) were Qatari nationals and 310 (25.6 %) were long-term residents. Approximately two-thirds of pilot participants were educated to graduate level or above.

**Conclusions:**

The pilot has proven that recruitment of volunteers into the Qatar Biobank project with intensive baseline measurements of behavioural, physical, and clinical characteristics is well accepted and logistically feasible. Qatar Biobank will provide a powerful resource to investigate the major determinants of ill-health and well-being in Qatar, providing valuable insights into the current and future public health burden that faces the country.

## Background

Over the past 50 years Qatar has experienced major economic growth and demographic and socio-economic changes. During this time the population of the country has risen rapidly, from 369,079 in 1986 [1], to over 2.4 million in 2015 (1.9 million aged over 15 years) [2] - most of this increase was driven by an influx of economic migrants, and there are estimated to be 300,000 Qatari nationals (~14 % of the total population). The population has experienced a major shift in diet with increasing availability of western type foods, and consequent increasing consumption of fat rich foods, meat and meat products, refined sugar, and industrially processed foods. In addition, similar to other high income countries, food has become relatively inexpensive compared to the average purchasing power. In parallel, changes in the economic, industrial, and urban landscape have led to a substantial reduction in physical activity, mirroring what has happened over the past decades in most of the economically developed world [3]. In this societal context, obesity has become increasingly prevalent in Qatar as it has in many countries around the world [4]. The 2012 national STEPwise survey reported that approximately 70 % of the population is overweight or obese (body mass index (BMI) ≥25 kg/m^2^) [3]. Obesity related comorbidities are now as common in Qatar as in most neighbouring countries, with the 2012 national STEPwise survey reporting high prevalence of hypertension (32.9 % of respondents ages 18-64) and diabetes (17.6 % of men and 15.9 % of women) [3]. As a comparison, the prevalence of hypertension across the UK adult population age 20–59 is around 15.0 % [5] and the prevalence of diabetes at all ages is 6.0 % [6]. Overall, in Qatar, chronic diseases such as cardiovascular diseases, diabetes, and cancer were estimated to account for 69 % of all deaths in 2008 [7].

Chronic diseases are caused by the complex interplay between environmental factors (such as diet, lifestyle, and the built environment) and genetic predisposition. To understand the aetiological role of environmental, behavioural, and genetic factors and their interactions, large-scale population cohorts have been established, mainly in Europe, North America, China, Japan, and Korea [8–16]. No such large population based studies currently exist in the Gulf Region. The Qatar Biobank was set up by the Qatar Foundation and the Supreme Council of Health in collaboration with Imperial College London, as the first Qatar national population based prospective cohort study, and includes the collection of biological samples, with long-term storage of data and samples for future research (biobank). Up to 60,000 men and women Qatari nationals and long-term residents (defined as individuals living in Qatar for 15 years and over) will be recruited into the cohort over the coming years, with extensive baseline clinical, metabolic and behavioural phenotypic data, and blood, urine, and saliva samples collected and stored. The Qatar Biobank will thus provide a powerful resource to investigate the role of environmental factors, lifestyle factors, genetics and their interactions in subsequent disease occurrence. As well as studying the causes of diseases, the Qatar Biobank will also provide insights into the current and future public health burden that faces the country.

Here we describe the design and methods of the Qatar Biobank study. The questionnaires, clinical measurements, biological sampling protocols and Standard Operating Procedures (SOPs) were developed during pre-plot testing and a pilot study, and we also provide here demographic and clinical referral information for the 1209 pilot study participants.

## Methods

**Fig. 1:**
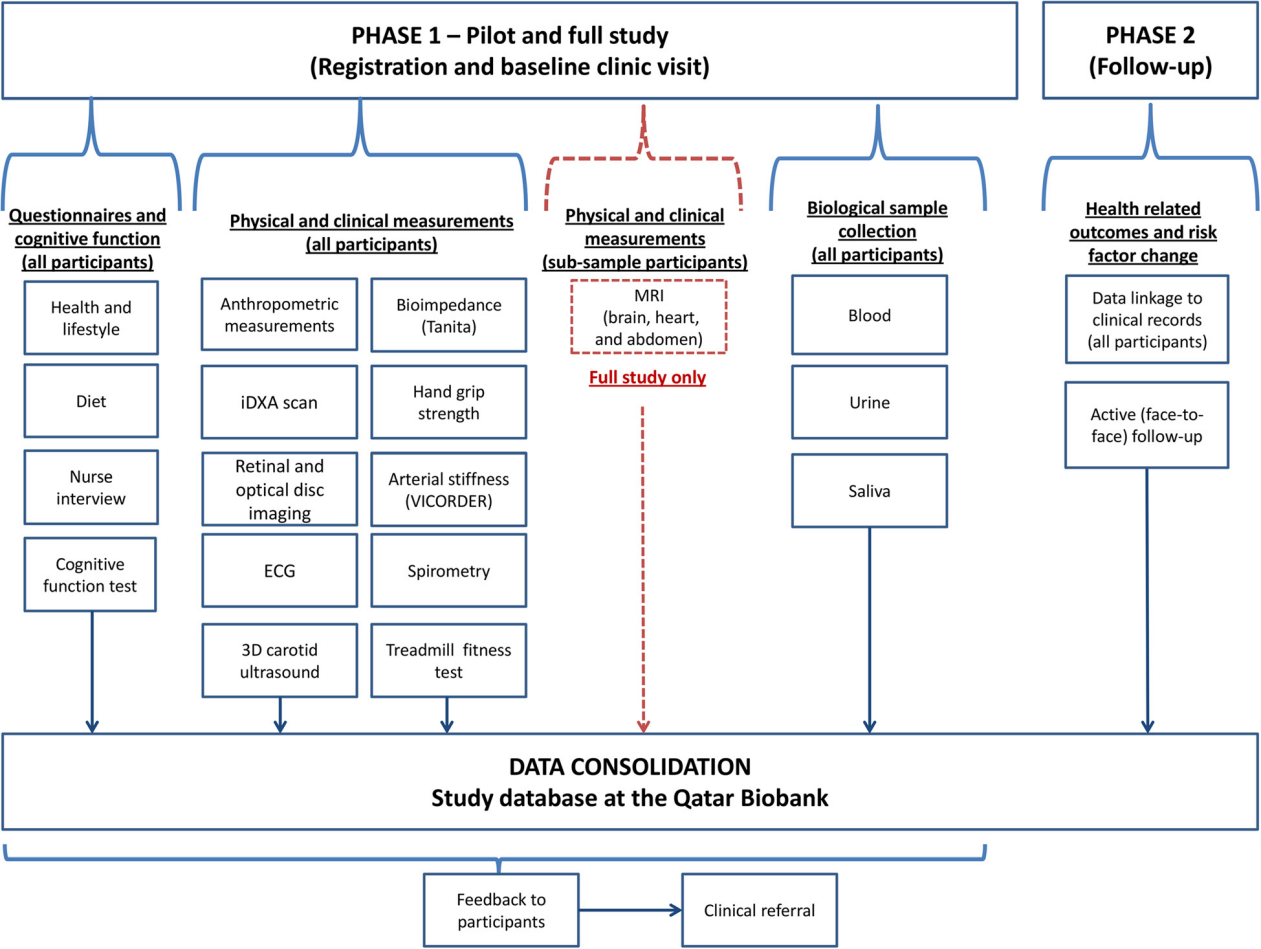
A summary of the data collected in the Qatar Biobank pilot and full study (including follow-up)

The Qatar Biobank involves collection of extensive questionnaire information, clinical phenotyping and biological samples from Qatari nationals and long-term residents (≥15 years living in Qatar) aged 18 or more years, who comprise the eligible population. A computerised clinic based system was developed for the pilot study to facilitate data collection, tracking of the participant data (and linked samples) throughout the visit and digital download from the clinical devices to minimise manual data entry. A summary of the data collected in the pilot study and for the full study (including follow-up) is shown in Fig. 1.

### Recruitment into the pilot study

For the full study, it is intended that participants will be broadly representative of the eligible Qatari population. However, for the pilot study, recruitment focused initially on selected occupational groups and word of mouth to provide a ready means to test the study protocols and SOPs. Recruitment to the pilot was initiated on the 11^th^ December 2012 and we report on data collected to 20^th^ February 2014.

**Fig. 2:**
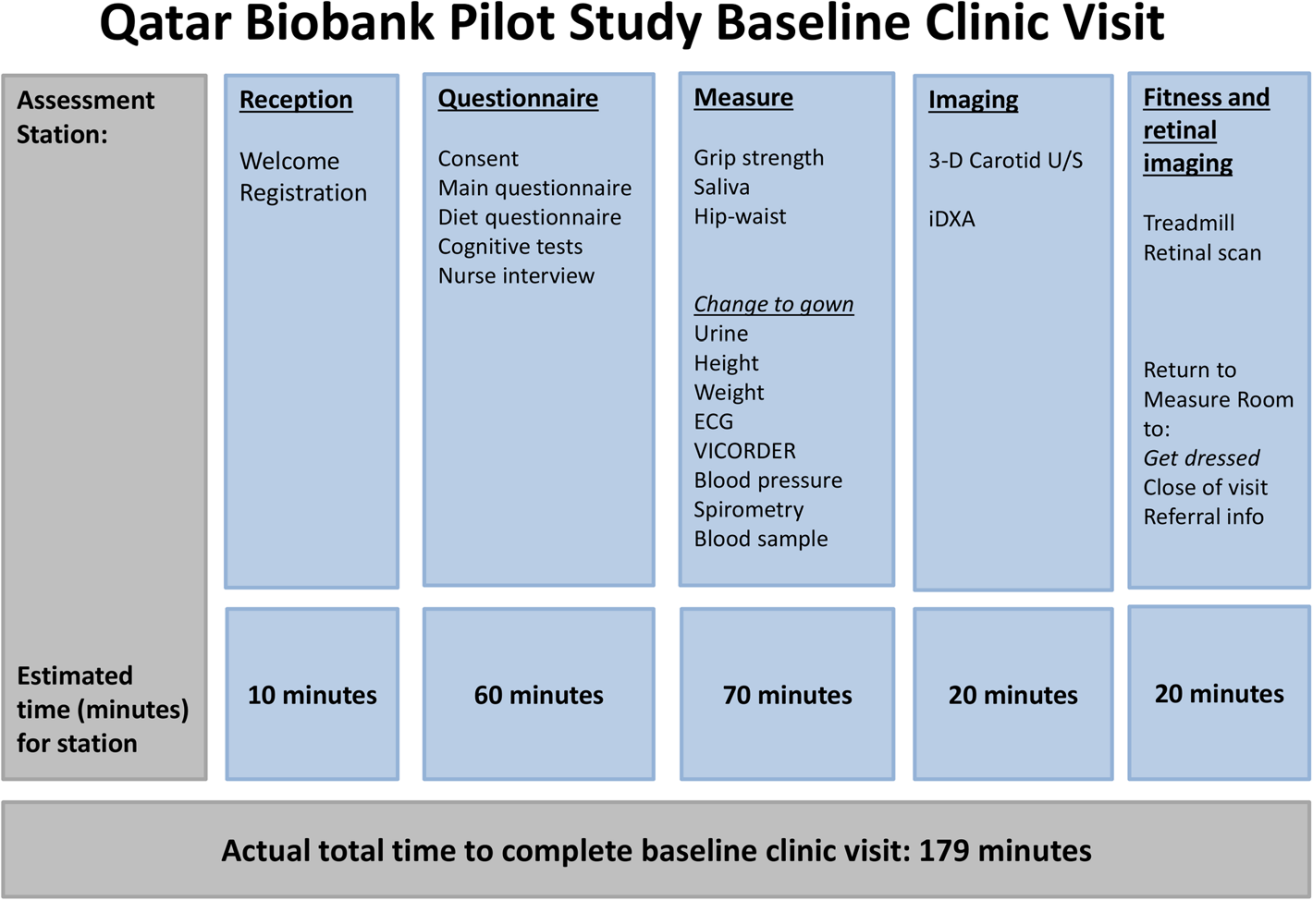
Summary of the Qatar Biobank pilot study baseline visit

For the pilot study, initially potential participants were contacted via a small number of public and private employers by setting up information booths at their workplace. Subsequently, most participants were recruited by personal recommendations of friends and family. At baseline, participants were invited to a visit at the Qatar Biobank facility at Hamad Medical City where they underwent a 5-stage interview and physical and clinic measurement sequence, with an average duration of 179 min (Fig. 2; Table 1). All participants gave informed consent. Institutional Review Board approval was obtained from the Hamad Medical Corporation Ethics Committee.

**Table 1:** Measurements in the Qatar Biobank pilot study

Measurement category	Instrument detail
*Questionnaires and cognitive function*	
Heath and lifestyle questionnaire	Questions on socio-demographic factors, current and past health conditions, current and past smoking habits (tobacco and shisha), occupation, mobile phone use, activity levels, sleeping patterns, and cognitive and psychological state
Diet questionnaire	Any past modifications to their diet and how often they consumed various foods and beverages over the preceding year
Nurse interview questionnaire	Previous or prevalent health conditions they or their family members may have suffered from; information on over-the-counter and prescription medications used and, in women only, reproductive factors
Cognitive function	Paired-associated learning questions to assess global cognition. Reaction time tests for touch screen administration
*Physical and clinical measurements*	
Anthropometry and body composition	Height, weight, waist circumference, and hip circumference Bioimpedance analysis (Tanita) Full body dual energy X-ray absorptiometry (iDXA) (GE) scan
Bone health	Full body dual energy X-ray absorptiometry (iDXA) (GE) scan
Grip strength	Grip strength using a Jamar J00105 hydraulic hand dynamometer
Retinal and disk imaging	Microscopic features of the optic nerve and macula assessed by using a Topcon TRC-NW6S retinal camera
Cardiovascular system	Blood pressure using the Omron 705 automated device (measured twice, or three times if first and second measurements differed by ≥5 mm Hg) Electrocardiogram (ECG) using the Atria 6100 automated system Arterial stiffness as assessed by VICORDER device 3D carotid ultrasound using a Philips ultrasound system and mechano‐transducer probe
Respiratory function	The Pneumotrac Vitalograph spirometry test
Cardiorespiratory fitness	Treadmill sub-maximal fitness test with heart rate monitoring
*Biological samples*	
Biological samples collected	Blood, urine, and saliva

### Self-completed health and lifestyle questionnaire

The design of the Qatar Biobank health and lifestyle questionnaire was informed by pre-pilot testing phases among Qatari volunteers, who provided information about the feasibility and acceptability of different versions of the questionnaire. The computer administered health and lifestyle section of the questionnaire contains detailed questions on socio-demographic factors, current and past health, family history of health conditions, current and past smoking habits (cigarettes and water pipe or shisha), occupational information, mobile phone use, physical activity levels, sleeping patterns, reproductive health (women), and cognitive and psychological state. Collection of information on cultural and lifestyle characteristics of the Qatari population for the pilot study was based on adaptation of widely used and validated instruments including from the European Prospective Investigation into Cancer and Nutrition (EPIC) study (smoking history, female reproductive history) [9], UK Biobank (e.g. sleep pattern questions) [10, 11], COSMOS (mobile phone use) [17, 18], the Patient Health Questionnaire (PHQ-9) (depression) [19], and the shortened form of the International Physical Activity Questionnaire (IPAQ) [20], which was augmented by questions on inactivity. In response to pre-pilot testing feedback, the health and lifestyle questions presented on the computer screen were tailored to the participant’s age, gender, and marital status. Similarly, a question skip pattern was applied to conditional questions as appropriate (e.g. non-smokers were not asked smoking history questions) and various logic and consistency checks were built into the software to reduce error rates. A trained nurse was available for participant assistance upon request.

### Dietary assessment

In the absence of an established questionnaire for assessment of diet in Qatar, the instrument used in Qatar Biobank was developed based on field assessment of the local food environment, focus groups, and consultation with local nutrition researchers. The computer-administered diet questionnaire assessed the intake of 96 food and beverage items, with either five or six frequency options depending on the nature of the item. The diet questionnaire also incorporated general questions on dietary habits, including reasons for recent dietary modification (if applicable), frequency of eating from a common plate, and snacking between meals. In order to assess the internal validity of the questionnaire, general questions on frequency of consuming broad categories of foods (chicken, meat, fish, fast and take-away foods, snacks, salads) were examined in relation to the sum of individual items within the broader categories (e.g. all fast food items), with Spearman’s rank correlations ranging from 0.30 (for sweet and savoury snacks) to 0.74 (for fish consumption).

### Cognitive function tests

Computer based self-administered tests were completed to assess cognitive function, specifically a choice reaction time test and a paired episodic memory test [21].

### Nurse administered interview questionnaire

In a face-to-face interview, administered by a trained nurse, participants were asked to report any previous or prevalent health conditions they or their family members may have suffered from; plus information on over-the-counter and prescription medication use and reproductive history (women only).

### Physical and clinical measurements

Various physical measurements were collected from each participant. Anthropometric measurements comprised body weight, height (sitting and standing) using the Seca stadiometer, hip and waist circumferences as well as bioimpedance (Tanita). To assess muscle strength, grip strength was measured in the participants’ right and left hands using a hydraulic hand dynamometer (Jamar J00105) [22]. Participants had an electrocardiogram (ECG) using the Atria 6100 automated system [23]. Arterial stiffness was assessed by the VICORDER device [24]. For blood pressure, using the Omron 705 automated device [25], two diastolic and systolic blood pressure measurements were obtained, and if these differed by 5 mmHg or more, a third measurement was made. Respiratory function was assessed by spirometry using the Pneumotrac Vitalograph [26].

Several imaging technologies were used. These included: 3D carotid ultrasound to measure intima media thickness (IMT) and carotid plaques using a Philips ultrasound system and mechano‐transducer probe [27]; full body dual energy X-ray absorptiometry (iDXA; General Electric) scan to assess bone mineral density and body composition [28]; and “microscopic” features of the optic nerve and macula assessed by use of a Topcon TRC-NW6S retinal camera [29]. Cardiorespiratory fitness was tested by a graded treadmill test of 5 to 11 min duration (dependent on self-rated fitness) using the h/p/cosmos quasar device [30]. For the full study, magnetic resonance imaging (MRI) of the brain, heart and abdomen is planned among a sub-sample of participants.

### Biological material collected

During the baseline recruitment visit, pilot participants provided samples of blood, saliva and urine. Approximately 60 ml of blood was collected from each participant. A proportion of the blood was used for the measurement of the 66 clinical biomarkers routinely measured (Table 2). Haematology and blood biochemistry were analysed by the laboratories of the Hamad Medical Centre Laboratory, Doha. The remainder, plus the urine and saliva, was subdivided into a number of aliquots, and then transferred into 2 dimensional barcode labelled microtubes for long-term cryogenic storage. A participant’s sample aliquots were split for storage in two separate locations—one for active use stored at −80 °C and one as a long-term backup (in liquid nitrogen vapour phase for the full study). The EDTA blood samples were centrifuged to separate blood into its constituent components, in the form of layers: plasma, buffy coat (leucocytes) and erythrocytes.

**Table 2:** The 66 clinical biomarkers routinely measured in the Qatar Biobank pilot study

Group	Variable
Bone and joint markers	Calcium
	Phosphorus
	Uric acid
	Vitamin D
Coagulation tests	Activated partial thromboplastin time
	Fibrinogen level
	International normalized ratio
	Prothrombin time
Diabetes related tests	C-Peptide
	Glucose
	Glycated Haemoglobin A1c %
	Insulin
Differential white cell count	Basophil
	Basophil %
	Eosinophils
	Eosinophils %
	Lymphocytes
	Lymphocytes %
	Monocyte
	Monocyte %
	Neutrophils
	Neutrophils %
	White blood cell
Electrolytes and renal function tests	Chloride
	Serum creatinine
	Bicarbonate
	Potassium
	Sodium
	Urea nitrogen
Full blood count	Haematocrit
	Haemoglobin
	Mean corpuscular haemoglobin
	Mean corpuscular HGB concentration
	Mean corpuscular volume
	Mean platelet volume
	Platelets
	Red blood cell
Sex steroid hormones	Estradiol
	Sex hormone binding globulin
	Testosterone
Inflammation/Autoimmune	Rheumatoid factor
	C-Reactive protein
Lipid profile	Cholesterol
	High density lipoprotein
	Low density lipoprotein
	Triglycerides
Liver function tests	Albumin
	Alkaline phosphatase
	Alanine transaminase
	Aspartate transaminase
	Gamma glutamyl transferase
	Total bilirubin
	Total protein
Minerals	Iron
	Ferritin
	Magnesium
	Total Iron binding capacity
Muscle markers	Creatine kinase
	Myoglobin
Thyroid function tests	Free triiodothyronine
	Free thyroxine
	Thyroid stimulating hormone
Vitamins	Vitamin B12
	Folate serum
Other tests	Homocysteine
	N-terminal brain-type natriuretic peptide

## Results

**Fig. 3:**
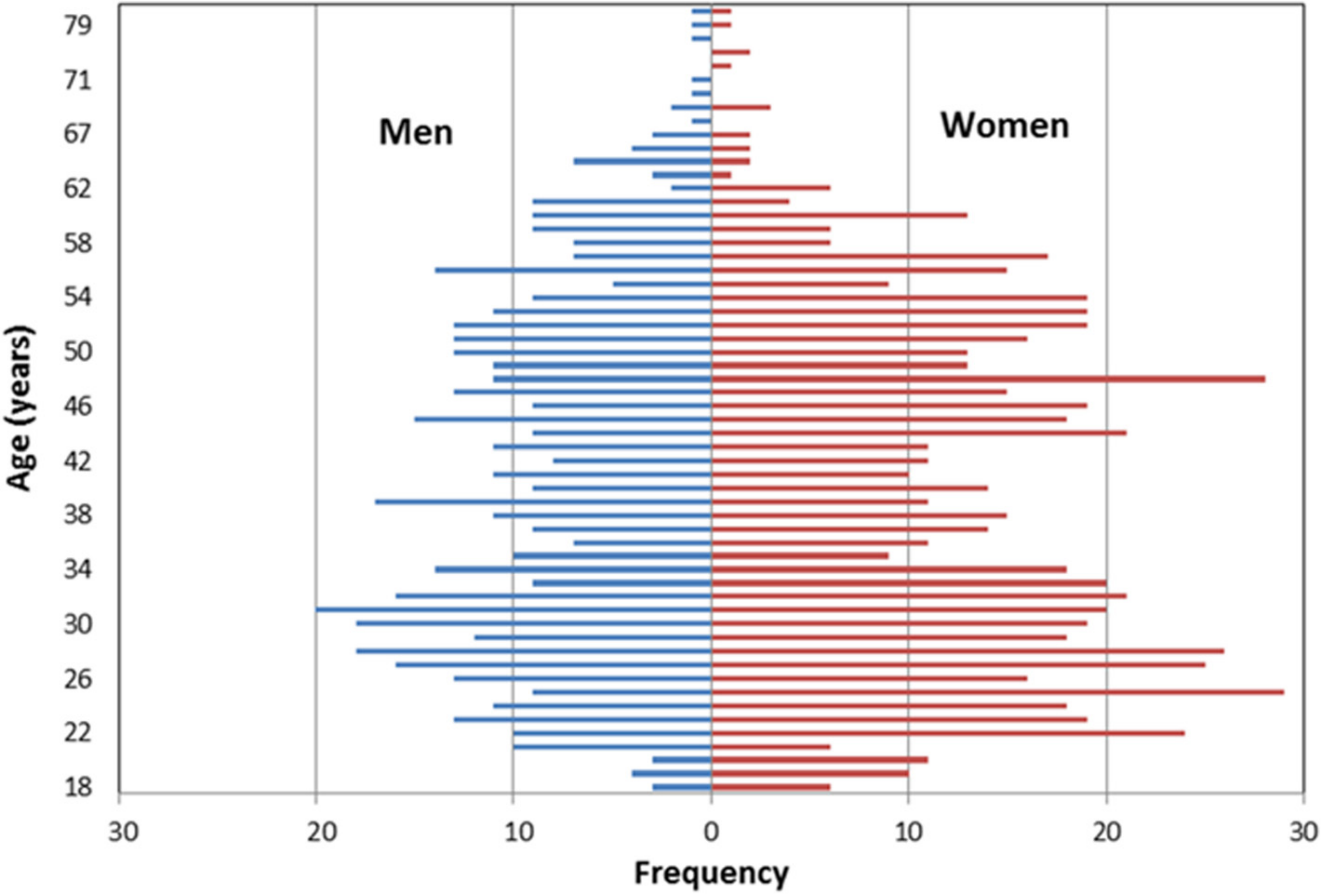
Age distribution of men and women recruited into the Qatar Biobank pilot study (*N* = 1209, mean = 39.89 years, SD = 12.92; median = 39 years)

Age of the 1209 participants recruited in the pilot study ranged between 25 and 80 years, with a median age of 39 years; 42 % were men, 58 % women (Fig. 3). The majority of participants were Qatari nationals (74 %) and 19 % were long-term residents of Arabic origin; the remaining 6 % were long-term residents of non-Arabic origin. Approximately two-thirds of men (67.6 %) and women (64.1 %) in the pilot study were educated to university graduate level and above (Table 3). Paid employee was the most common employment status for both men (74.5 %) and women (45.5 %).

**Table 3:** Baseline socio-demographic characteristics of participants in the Qatar Biobank pilot study (December 2012-February 2014 (*N* = 1209))

Characteristic	Men	Women	
	*N* (%)	*N* (%)	*P*-value
Age group (years)			0.15
< 25	54 (10.7)	94 (13.4)	
25–34	145 (28.7)	212 (30.2)	
35–44	102 (20.2)	127 (18.1)	
45–54	118 (23.3)	179 (25.5)	
≥ 55	87 (17.2)	91 (12.9)	
Marital status			<0.001
Married	402 (79.5)	461 (65.6)	
Divorced/separated	14 (2.7)	33 (4.7)	
Widow/widower	1 (0.2)	23 (3.3)	
Single	89 (17.6)	186 (26.4)	
Education			<0.001
Less than primary school	1 (0.2)	28 (3.9)	
Primary school	10 (2.0)	30 (4.3)	
Secondary school	36 (7.1)	37 (5.3)	
Technical/professional school	116 (22.9)	155 (22.1)	
University	270 (53.4)	408 (58.0)	
Postgraduate degree	72 (14.2)	43 (6.1)	
Missing	1 (0.2)	2 (0.3)	
Employment status			<0.001
Student or trainee	32 (6.3)	70 (10.0)	
Paid employee	377 (74.5)	320 (45.5)	
Self-employed/business owner	49 (9.7)	9 (1.3)	
Housewife	0 (0)	189 (26.9)	
Retired	22 (4.3)	80 (11.4)	
Unemployed	8 (1.6)	15 (2.1)	
None of the above	8 (1.6)	19 (2.7)	
Prefer not to answer	9 (1.8)	1 (0.1)	
Missing	1 (0.2)	0 (0)	

*P*-values for sex comparison calculated using Chi-squared test

Clinical referrals were made for participants with out of range values of clinical data, based on review by a doctor or a nurse. During the pilot study, 520 of the 1209 participants (43 %) were clinically referred. Most of these referrals were made for diabetes related tests (*N* = 198 participants; 16.3 %), low forced expiratory volume in one second based on spirometry (predicted FEV1 less than 80 % based on the best spirometry attempt, *N* = 173; 14.3 %), and indications of poor bone health based on iDXA scan measurement of bone density and low vitamin D levels (*N* = 160; 13.2 %).

## Discussion

The Qatar Biobank offers an unprecedented opportunity to study the causes and public health burden of diseases affecting the Qatari population which has undergone a major health transition over the past 50 years. It is collecting a broad range of phenotypic data. These include data from questionnaires, extensive clinical measurements and imaging, and biological samples (blood, urine, and saliva), which are being stored long-term, with consent, for future (unspecified) research use including for genetic studies. The pilot study was well accepted with high satisfaction levels reported by participants; 94 % (*N* = 1136) reported that if given the opportunity they would take part again. Following completion of the pilot, the study is now entering a phase of progressive increase in the number of participants attending the baseline visit, with recruitment widened to capture a sample designed to be as representative as possible of the eligible Qatari population, aiming for a total sample size of up to 60,000 individuals. A dedicated high-specification building has been assigned to the Qatar Biobank, including a clinic facility, laboratory, liquid nitrogen storage facility, offices for clinic and research staff, and an MRI suite. Data collection for the full study is planned over the next several years to achieve the projected sample size.

Already, the Qatar Biobank is having an impact on the health of participants due to the rigorous system of clinical referrals based on out of range values from the extensive clinical phenotypic information collected. Clinical measurements and biochemical data are fed back to participants by a doctor or nurse and clinical referrals (with consent) are made as necessary. Clinical referrals following face-to-face feedback of results to participants 3 to 6 weeks after their initial visit are an important and unique feature of the Qatar Biobank; such referrals have not been included in the protocol of most large-scale population based prospective studies. The feedback from study participants indicates that this process is highly valued. There was a high proportion of participants who were clinically referred in the pilot study, reflecting high prevalence of chronic conditions such as diabetes, low bone density and Vitamin D deficiency. The referral criteria for the extensive clinical phenotypic information collected are being further evaluated to optimise sensitivity and specificity of the referral procedure.

The Qatar Biobank will be a unique resource with large numbers of the eligible population enrolled. Additionally, the breadth and depth of phenotypic information and biological samples collected from participants is unparalleled by any other study in the Middle East and Asia. The imaging modules will be further augmented in the full study when a sub-sample of participants will undergo MRI scans of the brain, heart and abdomen. Again to our knowledge, such a comprehensive and state-of-the-art imaging protocol has not been implemented in large-scale population cohorts in the Middle East and Asia.

Participants will be followed up long-term through data linkage to clinical records (with consent) and occurrences of health related outcomes will be recorded. Furthermore, it is planned that participants will be re-contacted actively in the future for the collection of repeat phenotypic and medical condition information.

## Conclusion

The Qatar Biobank is a major new prospective cohort study in the Gulf region, with extensive data collection and storage of biological samples and linkage to health records for follow up. This will provide unprecedented opportunity to study the future health and disease burden as they evolve over the coming years among the Qatari population.

## Abbreviations

BMI: body mass index
ECG: electrocardiogram
IMT: intima media thickness
iDXA: dual energy X-ray absorptiometry
